# Adherence to colorectal cancer screening in a private health insurance center in Argentina from 2008 to 2022

**DOI:** 10.17843/rpmesp.2024.414.13680

**Published:** 2024-10-25

**Authors:** Sebastián Sguiglia, Camila Volij, Manuel Rodríguez-Tablado, Sergio Terrasa, Santiago Esteban

**Affiliations:** 1 Hospital Italiano de Buenos Aires, Buenos Aires, Argentina. Hospital Italiano de Buenos Aires Buenos Aires Argentina; 2 University of the Hospital Italiano de Buenos Aires, Buenos Aires, Argentina. University of the Hospital Italiano de Buenos Aires Buenos Aires Argentina; 3 National Scientific and Technical Research Council, Buenos Aires, Argentina. National Scientific and Technical Research Council National Scientific and Technical Research Council Buenos Aires Argentina; 4 Institute of Clinical and Health Effectiveness, Buenos Aires, Argentina. Institute of Clinical and Health Effectiveness Buenos Aires Argentina

**Keywords:** Colonic Neoplasms, Mass screening, Secondary prevention, Occult blood, Colonoscopy, Argentina

## Abstract

In order to evaluate adherence to colorectal cancer (CRC) screening among members of the Health Plan of the Hospital Italiano de Buenos Aires in Argentina, we conducted a retrospective cohort study using secondary data from the electronic medical record. We included all members over 50 years of age during the period 2008-2022. We assessed the number and type of screening tests performed and the proportion of members covered for screening. We analyzed 112,112 participants, with a median age of 58.6 years and a follow-up time of 8.6 years. Colonoscopy was the most commonly used test. The maximum coverage reached was 47.1% in December 2022. In conclusion, adherence to CRC screening was suboptimal, as was the method used. This information can be used for the design of a multicomponent intervention.

## INTRODUCTION

Colorectal cancer (CRC) is one of the leading causes of morbidity and mortality in the world, and Argentina is one of the Latin American countries with the highest burden of disease, with an incidence and mortality of 24.2 cases and 12.2 deaths per 100,000 people per year, respectively ^1^^,^^2^.

CRC screening, by means of immunochemical fecal occult blood test (FOBT), is an efficient and cost-effective strategy to reduce mortality from this disease ^3^^,^^4^. The National Cancer Institute (INC) of Argentina recommends offering it to the general population between 50 and 75 years of age and considers 70% coverage to be desirable ^2^^,^^5^. Although the INC does not recommend videocolonoscopy (VCC) as a screening test in the general population, associations such as the United States Preventive Services Task Force (USPSTF) accept it as an alternative ^2^^,^^3^.

There are few published studies on adherence to CRC screening in the Argentine population ^6^^-^^9^. According to the National Survey of Risk Factors, conducted in 2018, 31.6% of individuals aged 50-75 years had this screening once in their lifetime. This proportion increases in individuals with higher educational or socioeconomic level, with social security or private health insurance coverage and in some jurisdictions such as the Autonomous City of Buenos Aires (CABA) ^6^.

Hospital Italiano de Buenos Aires (HIBA) is a network of two hospitals and fifteen outpatient centers located in CABA and Greater Buenos Aires (GBA). It operates as a provider for private insurance and social security and also offers health insurance (PS-HIBA) for which it is the sole provider. PS-HIBA members are predominantly middle class. We do not have an organized CRC screening program at the institution. Although our team has already published research related to this problem, we were not aware of the adherence to this practice in the PS-HIBA population ^10^^-^^13^. Therefore, this research aims to evaluate the adherence of PS-HIBA members to CRC screening.

**KEY MESSAGES**

Motivation for the study. There is a need for information on population adherence to colon cancer screening.

Main findings. Adherence to screening in health insurance increased to a maximum of 47.1% in December 2022, which is below desirable targets. The most commonly used method was colonoscopy.

Public health implications. This information could contribute to the design of a multicomponent intervention to improve adherence to colon cancer screening.

## THE STUDY

We conducted a retrospective cohort study with secondary data from electronic medical records (EMR). We included individuals aged 50 to 75 years who were affiliated for at least one month with PS-HIBA between January 1, 2008 and December 31, 2022. We excluded patients with missing or discordant data regarding their affiliation period or with risk factors for developing CRC (personal or family history of CRC, colonic polyps, inflammatory bowel disease, hereditary CRC syndrome) or a total colectomy performed before entering the cohort.

A list of the individuals who met the inclusion criteria was requested from the Information Management Area of the HIBA Research Department, together with their dates of birth, PS-HIBA affiliation, disaffiliation or death, place of residence, and sex. Risk factors were identified using a subset of problems associated with risk factors for colon cancer or colectomy loaded into the EMR (appendix 1). We requested information about studies (SOMF or VCC) performed by participants during follow-up from the same source. We reviewed a random sample of 100 medical records for completeness and accuracy of the database.

Participants entered the cohort when they met the inclusion criteria (age over 50 years or affiliation to PS-HIBA) and remained in the cohort until their disenrollment date, death, until they reached 75 years of age, or until the end of the study (12/31/2022). The main outcome of interest was screening coverage, i.e. what proportion of participants were covered at the end of each month, having undergone a FOBT in the previous year or a VCC in the previous 10 years.

In addition, the number of screening studies performed per year by members of PS-HIBA and the proportion of them who performed at least one study during the observation period and the annual rate of screening studies performed, dividing, for each year, the total number of studies performed by the average number of active members.

As secondary outcomes, we measured the proportion of FOBT with a positive result (positivity rate), the proportion of VCCs with abnormal results (polyps or other CRC precursor lesions) or insufficient preparation, the proportion of FOBT-positive patients who had a VCC within six months of the result, the proportion of VCCs preceded by a positive FOBT, and the proportion of electronic FOBT and VCC requests that were fulfilled by participants after six months (order length time).

We conducted a subgroup analysis to assess whether screening coverage at the end of follow-up was associated with age, sex, year of cohort entry, or place of residence. Chi-square tests and Cuzick’s nonparametric trend test were used for comparisons ^14^. As a sensitivity analysis, 2-year coverage for FOBT was considered. In order to assess the proportion of CCVs with abnormal results, we analyzed a simple random sample of 100 colonoscopies. All analyses and graphs were performed with R (R Foundation for Statistical Computing, Vienna).

This study was conducted with anonymized secondary data obtained from electronic medical records, in accordance with the Code of Ethics of the World Medical Association (Declaration of Helsinki). Approval was obtained from the HIBA Research Protocols Ethics Committee (code 1522).

## FINDINGS

The initial database included 114,763 participants, of which 2637 (2.3%) were excluded for having risk factors for CRC, 121 (0.1%) had missing or contradictory data during their affiliation periods, and 14 for previous colectomy. The final size was 112,112 participants.

**Figure 1:**
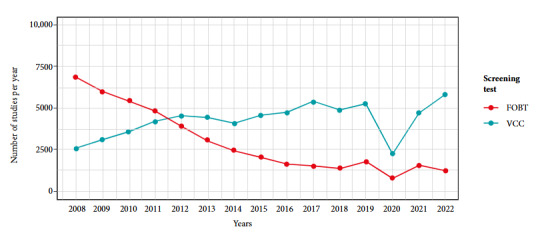
Colon cancer screening tests performed between 2008 and 2022 by members of the Health Plan of the Hospital Italiano de Buenos Aires in patients between 50 and 75 years of age.

Table 1 shows the characteristics of the population enrolled in the Health Plan of the Hospital Italiano de Buenos Aires, Argentina, between 2008 and 2022. The median age at admission was 58.6 years (interquartile range: 50.8 to 65.8) and the median follow-up time was 8.6 years (interquartile range: 3.6 to 14.6). A total of 107,495 screening tests were recorded during the observation period, of which 63,603 (59.2%) corresponded to VCC and 43892 (40.8%) to FOBT. Figure 1 shows the temporal evolution of the performance of these tests. In 2022, the rates of FOBT and VCC were 21.8 and 105.3 studies per 1,000 members per year, respectively. Of the participants, 52.4% underwent at least one screening test, with CCV being the most commonly used in 57.5% of cases, followed by a combination of both tests in 22.6% and FOBT in 19.8%.

**Table 1:** Characteristics of members between 2008 and 2022 of the Health Plan of the Hospital Italiano de Buenos Aires, Argentina.

Variable		n (%)
Sex		
	Female	68397 (60.8)
	Male	44091 (39.2)
	Undetermined	3 (0)
Place of residence		
	City of Buenos Aires	50572 (45.0)
	Greater Buenos Aires	33433 (29.7)
	Rest of Argentina	1604 (1.4)
	No data	26882 (23.9)
Age at cohort entry		
	50 years	25068 (22.3)
	Over 50 years	87423 (77.7)

FOBT: Fecal Occult Blood Test immunological fecal occult blood test. VCC: Videocolonoscopy.

**Figure 2:**
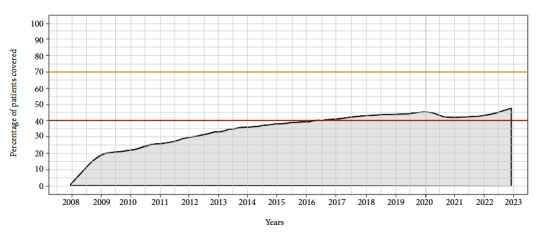
Coverage for colon cancer screening as a function of time Affiliates of the Health Plan of the Italian Hospital of Buenos Aires. Period 2008-2022.

Figure 2 shows the evolution of coverage over time. In December 2022, 47.1% of participants were covered, which represents the maximum coverage rate achieved during the study. Sex, age older than 60 years, place of residence in CABA or GBA, year of entry into the cohort before 2017, and use of colonoscopy as a screening method were associated with higher coverage (Table 2).

FOBT: Immunological fecal occult blood test. VCC: Videocolonoscopy. Proportion of members of the Hospital Italiano de Buenos Aires Health Plan aged between 50 and 75 years, who are covered for colorectal cancer screening at the end of each month of follow-up, having undergone a FOBT in the previous year or a VCC in the last ten years. The red and gold horizontal lines represent 40% and 70% coverage, which are the targets considered acceptable and desirable, respectively, by the National Cancer Institute of Argentina. For the first years of follow-up, the level of coverage may be underestimated, because screening studies performed prior to 2008 were not included in the database.

**Table 2:** Coverage for colon cancer screening achieved by December 2022. Subgroup analysis.

Subgroup		Participants covered for colon cancer screening (%)	Participants without coverage for colon cancer screening (%)	p-value
Sex				
	Male	9962 (46.0)	11,686 (54.0)	0.001^e^
	Female	14,846 (47.8)	16,239 (52.2)	
Age ^a^				
	50-55	2014 (24.1)	6350 (75.9)	0.001^f^
	51-60	4548 (43.1)	6006 (56.9)	
	61-65	5426 (51.2)	5180 (48.8)	
	66-70	5940 (54.3)	4990 (45.7)	
	71-75	6880 (56.0)	5399 (44.0)	
Age (dichotomous)				
	50-60	6562 (34.7)	12,356 (65.3)	0.001^e^
	61 or more	18,246 (54.0)	15,569 (46.0)	
Place of residence				
	CABA	11,387 (49.1)	11,828 (50.9)	0.001^e^
	GBA	7280 (45.7)	8666 (54.3)	
	Rest of Argentina	279 (40.3)	414 (59.7)	
Year of cohort entry				
	2008-2017 ^b^	20,717 (54.8)	17,098 (45.2)	0.001^e^
	2018-2022	4091 (27.4)	10,827 (72.6)	
Screening method used ^c^				
	FOBT	946 (23.1)	3156 (76.9)	0.001^e^
	VCC	18,369 (95.1)	944 (4.9)	
	Combination of FOBT and VCC	5493 (89.7)	628 (10.3)	
Follow-up completion				
	Participants with residence in CABA or GBA, who entered the cohort at age 50 and before 2017 ^d^	4660 (52.4)	4238 (47.6)	0.001^e^
	Rest of the participants	2048 (46.0)	23,687 (54.0)	

a) Age refers to age in years at the time of evaluation (December 2022). b) These patients have at least 5 years of follow-up. c) Refers to the screening tests used by the participants during the entire follow-up period, not necessarily the last test performed. d) We present the results for this subgroup separately since it is less likely to have performed screening studies before entering the cohort (due to age) and to have performed them outside the PS-HIBA care network (due to place of residence). In addition, they have a follow-up time of at least 5 years. e) Chi-square test. f) Cuzick nonparametric trend test. CABA: Autonomous City of Buenos Aires. GBA: Greater Buenos Aires. FOBT: Fecal occult blood test. VCC: Videocolonoscopy.

The FOBT positivity rate was 17.1%. Of the participants with positive FOBT, 50.2% had a CCV within six months. On the other hand, only 12.6% of the CCVs were performed after a positive FOBT.

Forty-three percent of CCVs had an abnormal result (95% CI: 33.1% - 53.2%) and 4% had inadequate preparation (95% CI: 1.1% - 9.9%). Of the electronic FOBT and VCC requests, 52.5% and 49.1% were effective within 6 months of request, respectively.

In the sensitivity analysis, considering a two-year coverage for FOBT, the proportion of patients covered for screening in December 2022 was slightly higher, with a value of 48.4% (p<0.001).

## DISCUSSION

Our findings show that in December 2022 the coverage was 47.1%, which represents the maximum coverage achieved within the study period. CCV was the most widely used method, with a rate per year of 105.3 studies per 1000 members.

During 2020 we found a marked decrease in the number of studies, this could be attributable to the isolation measures by COVID-19 ^15^. During 2018, a relative decrease in the performance of VCC was also found, which could be explained by the death of a well-known Argentine journalist during an endoscopy; while in 2019 there was a rise in the number of FOBT, attributable to the replacement in our institution of the guaiac method for identifying occult blood in fecal matter by immunochemical FOBT, which is simpler for the patient ^16^.

The level of achieved coverage is lower than the 70% target proposed by the INC. In addition, the most widely used test was VCC instead of FOBT, which represents the method of choice according to the INC ^2^^,^^5^.

According to the 2018 National Risk Factor Survey, the proportion of people aged 50-75 years who had ever been screened for colon cancer was 51.3% in CABA, and the most commonly used method was CCV in 42.6% of cases, which is similar to the results of our study ^6^.

Regarding the causes of low coverage, two studies have been published in Argentina that point to the low knowledge of the population about CRC and screening methods, particularly the FOBT, the low perception of risk and cultural taboos related to the anus, as barriers to access to screening ^17^^,^^18^. Other factors that may affect coverage are adverse economic circumstances, weakness or fragmentation of the health system, and the absence of an organized screening program that reaches the entire population ^19^.

The study has limitations; we were unable to identify from our database whether patients participated in screening outside HIBA or prior to cohort entry. It is unlikely that this happened in the subgroup of individuals entering the cohort at 50 years of age and residing in CABA or GBA, so our confidence in the coverage estimate for this subgroup is higher. On the other hand, we do not have information on the reason for performing the studies.

However, as proposed by Chubak and Hubbard, we considered patients as covered, i.e. without the need for new screening tests, if they underwent a FOBT in the last year or a VCC in the last ten years, regardless of the reason for performing them ^20^. We also did not take into account the outcome of the studies in the calculation of coverage. If we consider that 17% of the FOBT were positive and 43% of the CCVs had abnormal findings at endoscopy, the real coverage of the affiliates is probably lower. Finally, as this is a single-center study, our results cannot be extrapolated to the general population of Argentina or other countries in the region.

In terms of strengths, to the best of our knowledge, this is the first study that evaluates adherence to CRC screening in the Argentine private health subsystem. We included all members belonging to the target population according to age range. The use of EMR data allows a longitudinal registry and follow-up of the participants.

In conclusion, CRC screening coverage is far from desirable levels, despite certain favorable characteristics of PS-HIBA, such as the socioeconomic level of the population and the high availability of CCV. This information can be useful as a basis for the design of a multicomponent intervention that contributes to increasing adherence to CRC screening, which should focus on the knowledge and accessibility of the FOBT in both patients and health professionals.
