# Supplementary material for: Adherence to colorectal cancer screening in a private health insurance center in Argentina from 2008 to 2022
Source: Rev Peru Med Exp Salud Publica. 2024 Oct 25;41(4):405–10. doi: 10.17843/rpmesp.2024.414.13680 (PMC11797579; doi:10.17843/rpmesp.2024.414.13680)
Supplement: Supplementary material. — Available in the electronic version of the RPMESP. [file rpmesp-41-04-13680-s001.docx]

**Materiales suplementarios:**

**Anexo 1.** *Subset* de problemas asociados con factores de riesgo para cáncer de colon o colectomía total

| Categoría | Entrada |
| --- | --- |
| Colectomía | Colectomía subtotal con ileocoloanastomosis |
| Colectomía | Colectomía subtotal e ileosigmoideo anastomosis |
| Colectomía | Colectomía total |
| Colectomía | Colectomía total con ileorrectoanastomosis |
| Colectomía | Colectomía total con ileostomía |
| Colectomía | Colectomía total con ileostomía terminal |
| Colectomía | Colectomía total e ileocoloanastomosis |
| Colectomía | Colectomía total laparoscópica |
| Colectomía | Proctocolectomia total |
| Cáncer de colon | Cáncer colorrectal |
| Cáncer de colon | Cáncer colorrectal metastásico |
| Cáncer de colon | Cáncer de colon |
| Cáncer de colon | Cáncer de colon ascendente |
| Cáncer de colon | Cáncer de colon avanzado |
| Cáncer de colon | Cáncer de colon con metástasis hepática |
| Cáncer de colon | Cáncer de colon derecho estadio 3 |
| Cáncer de colon | Cáncer de colon descendente |
| Cáncer de colon | Cáncer de colon descendente oclusivo |
| Cáncer de colon | Cáncer de colon en tratamiento quimioterápico |
| Cáncer de colon | Cáncer de colon estadio 1 |
| Cáncer de colon | Cáncer de colon estadio 3 |
| Cáncer de colon | Cáncer de colon estadio 3c |
| Cáncer de colon | Cáncer de colon estadio 4 |
| Cáncer de colon | Cáncer de colon estadio 4 con metástasis hepáticas y peritoneales |
| Cáncer de colon | Cáncer de colon krukenberg estadio 4 |
| Cáncer de colon | Cáncer de colon oclusivo |
| Cáncer de colon | Cáncer de colon perforado |
| Cáncer de colon | Cáncer de colon progresado |
| Cáncer de colon | Cáncer de colon recidivado |
| Cáncer de colon | Cáncer de colon sigmoides |
| Cáncer de colon | Cáncer de colon sigmoides con colostomía |
| Cáncer de colon | Cáncer de colon sigmoides estadio 3 |
| Cáncer de colon | Cáncer de colon sigmoides oclusivo |
| Cáncer de colon | Cáncer de colon sigmoides perforado |
| Cáncer de colon | Cáncer de colon sigmoides y recto |
| Cáncer de colon | Cáncer de colon transverso |
| Cáncer de colon | Cáncer de colon transverso oclusivo |
| Cáncer de colon | Cáncer de colon y hepatectomía derecha |
| Cáncer de colon | Cáncer de la unión rectosigmoidea |
| Cáncer de colon | Cáncer de recto |
| Cáncer de colon | Cáncer de recto bajo |
| Cáncer de colon | Cáncer de recto con metástasis hepáticas |
| Cáncer de colon | Cáncer de recto estadio 1 |
| Cáncer de colon | Cáncer de recto estadio 4 |
| Cáncer de colon | Cáncer de recto medio |
| Cáncer de colon | Cáncer de recto oclusivo |
| Cáncer de colon | Cáncer del ángulo esplénico del colon |
| Cáncer de colon | Cáncer del ángulo hepático del colon |
| Cáncer de colon | Cirugía de cáncer de colon |
| Cáncer de colon | Colectomía derecha por cáncer de colon |
| Cáncer de colon | Colectomía por cáncer de colon |
| Cáncer de colon | Estado posoperatorio por cirugía de cáncer de colon |
| Cáncer de colon | Hemicolectomía derecha por cáncer de colon |
| Cáncer de colon | Hemicolectomía derecha por neoplasia maligna primaria colon |
| Cáncer de colon | Hemicolectomía izquierda por cáncer colon |
| Cáncer de colon | Metástasis hepática por cáncer de colon |
| Cáncer de colon | Metástasis pulmonar por cáncer de colon |
| Cáncer de colon | Neoplasia maligna primaria de colon |
| Cáncer de colon | Neoplasia maligna primaria de colon descendente |
| Cáncer de colon | Operación de neoplasia maligna primaria del recto |
| Cáncer de colon | Pólipo colónico con transformación maligna |
| Cáncer de colon | Recaída de cáncer de recto |
| Cáncer de colon | Resección anterior alta por cáncer de recto |
| Cáncer de colon | Resección de cáncer de colon |
| Cáncer de colon | Resección de cáncer del recto superior |
| Cáncer de colon | Resección de neoplasia maligna de colon |
| Antecedente de cáncer en familiar de 1° grado | Antecedente familiar de cáncer colon en padre biológico |
| Antecedente de cáncer en familiar de 1° grado | Antecedente familiar de cáncer colorrectal en hermana natural |
| Antecedente de cáncer en familiar de 1° grado | Antecedente familiar de cáncer colorrectal en hermano biológico |
| Antecedente de cáncer en familiar de 1° grado | Antecedente familiar de cáncer colorrectal en madre biológica |
| Antecedente de cáncer en familiar de 1° grado | Antecedente familiar de cáncer colorrectal en padre biológico |
| Antecedente de cáncer en familiar de 1° grado | Antecedente familiar de cáncer de colon en familiar consanguíneo de primer grado |
| Antecedente de cáncer en familiar de 1° grado | Antecedente familiar de cáncer de colon en hermana biológica |
| Antecedente de cáncer en familiar de 1° grado | Antecedente familiar de cáncer de colon en hermana biológica fallecida |
| Antecedente de cáncer en familiar de 1° grado | Antecedente familiar de cáncer de colon en hermano biológico |
| Antecedente de cáncer en familiar de 1° grado | Antecedente familiar de cáncer de colon en hermano biológico fallecido |
| Antecedente de cáncer en familiar de 1° grado | Antecedente familiar de cáncer de colon en hija biológica |
| Antecedente de cáncer en familiar de 1° grado | Antecedente familiar de cáncer de colon en hijo biológico |
| Antecedente de cáncer en familiar de 1° grado | Antecedente familiar de cáncer de colon en madre biológica |
| Antecedente de cáncer en familiar de 1° grado | Antecedente familiar de cáncer de colon en madre biológica fallecida |
| Antecedente de cáncer en familiar de 1° grado | Antecedente familiar de cáncer de colon en padre biológico |
| Antecedente de cáncer en familiar de 1° grado | Antecedente familiar de cáncer de colon en padre biológico fallecido |
| Antecedente de cáncer en familiar de 1° grado | Antecedente familiar de cáncer de recto en hermana biológica |
| Antecedente de cáncer en familiar de 1° grado | Antecedente familiar de cáncer de recto en hermano biológico |
| Antecedente de cáncer en familiar de 1° grado | Antecedente familiar de cáncer de recto en madre biológica |
| Antecedente de cáncer en familiar de 1° grado | Antecedente familiar de cáncer de recto en padre biológico |
| Antecedente de cáncer en familiar de 1° grado | Antecedente familiar de neoplasia maligna primaria de colon en madre biológica |
| Antecedente de cáncer en familiar de 2° grado | Antecedente familiar de cáncer colon en abuelo paterno |
| Antecedente de cáncer en familiar de 2° grado | Antecedente familiar de cáncer colorrectal en abuelo paterno |
| Antecedente de cáncer en familiar de 2° grado | Antecedente familiar de cáncer de colon en abuela |
| Antecedente de cáncer en familiar de 2° grado | Antecedente familiar de cáncer de colon en abuela materna |
| Antecedente de cáncer en familiar de 2° grado | Antecedente familiar de cáncer de colon en abuela paterna |
| Antecedente de cáncer en familiar de 2° grado | Antecedente familiar de cáncer de colon en abuelo |
| Antecedente de cáncer en familiar de 2° grado | Antecedente familiar de cáncer de colon en abuelo materno |
| Antecedente de cáncer en familiar de 2° grado | Antecedente familiar de cáncer de colon en abuelo paterno |
| Antecedente de cáncer en familiar de 2° grado | Antecedente familiar de cáncer de colon en tía |
| Antecedente de cáncer en familiar de 2° grado | Antecedente familiar de cáncer de colon en tío |
| Antecedente de cáncer en familiar de 2° grado | Antecedente familiar de neoplasia maligna primaria de colon en abuelo materno |
| Síndrome de cáncer de colon hereditario | Cáncer de colon familiar |
| Síndrome de cáncer de colon hereditario | Poliposis adenomatosa de colon |
| Síndrome de cáncer de colon hereditario | Síndrome de Lynch |
| Enfermedad inflamatoria intestinal | Cirugía de colitis ulcerosa |
| Enfermedad inflamatoria intestinal | Colitis ulcerosa |
| Enfermedad inflamatoria intestinal | Colitis ulcerosa inespecífica |
| Enfermedad inflamatoria intestinal | Colitis ulcerosa reagudizada |
| Enfermedad inflamatoria intestinal | Enfermedad de Crohn |
| Enfermedad inflamatoria intestinal | Enfermedad de Crohn localizada en intestino delgado |
| Enfermedad inflamatoria intestinal | Proctocolitis ulcerosa |
| Antecedente personal de pólipo | Adenoma colónico |
| Antecedente personal de pólipo | Adenoma de colon con displasia de bajo grado |
| Antecedente personal de pólipo | Adenoma de colon displásico |
| Antecedente personal de pólipo | Adenoma tubular colónico |
| Antecedente personal de pólipo | Adenoma tubular colónico displasia |
| Antecedente personal de pólipo | Adenoma tubular de colon con displasia de alto grado |
| Antecedente personal de pólipo | Adenoma tubular de colon con displasia de bajo grado |
| Antecedente personal de pólipo | Adenoma tubular de colon descendente con displasia de bajo grado |
| Antecedente personal de pólipo | Adenoma tubulovelloso de colon |
| Antecedente personal de pólipo | Adenoma tubulovelloso de colon con displasia de alto grado |
| Antecedente personal de pólipo | Adenoma tubulovelloso de colon con displasia de bajo grado |
| Antecedente personal de pólipo | Adenoma tubulovelloso de colon derecho con displasia de bajo grado |
| Antecedente personal de pólipo | Adenoma velloso colon derecho |
| Antecedente personal de pólipo | Adenoma velloso colon izquierdo |
| Antecedente personal de pólipo | Adenoma velloso colon sigmoides |
| Antecedente personal de pólipo | Adenoma velloso de colon con displasia de alto grado |
| Antecedente personal de pólipo | Adenoma velloso del colon |
| Antecedente personal de pólipo | Adenomas tubulares colónicos |
| Antecedente personal de pólipo | Adenomas tubulares colónicos con displasia de bajo grado |
| Antecedente personal de pólipo | Anemia ferropénica por pólipo colónico |
| Antecedente personal de pólipo | Anemia ferropénica secundaria adenoma colon |
| Antecedente personal de pólipo | Diverticulosis colónica y pólipo colónico |
| Antecedente personal de pólipo | Polipectomía de adenoma de colon |
| Antecedente personal de pólipo | Pólipo adenomatoso de colon |
| Antecedente personal de pólipo | Pólipo colónico |
| Antecedente personal de pólipo | Pólipo colónico adenomatoso tubular con displasia de bajo grado |
| Antecedente personal de pólipo | Pólipo colónico adenomatoso tubulovelloso con displasia de bajo grado |
| Antecedente personal de pólipo | Pólipo colónico con displasia de alto grado |
| Antecedente personal de pólipo | Pólipo colónico con displasia de bajo grado |
| Antecedente personal de pólipo | Pólipo colónico hiperplásico |
| Antecedente personal de pólipo | Pólipos colónicos 2 a |
| Antecedente personal de pólipo | Pólipos colónicos con displasia de alto grado |
| Antecedente personal de pólipo | Pólipos colónicos displásicos |
| Antecedente personal de pólipo | Pólipos colónicos hiperplásicos |
| Antecedente personal de pólipo | Pólipos colónicos múltiples |
| Antecedente personal de pólipo | Poliposis adenomatosa tubular de colon |
| Antecedente personal de pólipo | Resección de pólipo sésil colónico |
| Antecedente personal de pólipo | Resección endoscópica de pólipo colónico |
| Antecedente personal de pólipo | Videocolonoscopia con resecado de adenoma tubulovelloso colónico |
| Antecedente familiar de pólipo | Antecedente familiar de pólipo colónico en hermano biológico |
| Antecedente familiar de pólipo | Antecedente familiar de pólipo colónico en madre biológica |
| Antecedente familiar de pólipo | Antecedente familiar de pólipos colónicos en madre biológica |

| **Anexo 2.** 24 Partidos de la Provincia de Buenos Aires incluidos en el Gran Buenos Aires   \| Almirante Brown, Avellaneda, Berazategui, Esteban Echeverría, Ezeiza, Florencio Varela, General San Martín, Hurlingham, Ituzaingó, José C. Paz, La Matanza, Lanús, Lomas de Zamora, Malvinas Argentinas, Merlo, Moreno, Morón, Quilmes, San Fernando, San Isidro  San Miguel, Tigre, Tres de Febrero, Vicente López \| \| --- \| |  |
| --- | --- | --- |
|  |  |
