# Supplementary material for: Adherence to colorectal cancer screening in a private health insurance center in Argentina from 2008 to 2022
Source: Rev Peru Med Exp Salud Publica. 2024 Oct 25;41(4):405–10. doi: 10.17843/rpmesp.2024.414.13680 (PMC11797579; doi:10.17843/rpmesp.2024.414.13680)
Supplement: Translation

# Translation: Adherencia al tamizaje de cáncer colorrectal en un seguro privado de salud de Argentina entre 2008 y 2022

**Authors:** Sebastián Sguiglia, Camila Volij, Manuel Rodríguez-Tablado, Sergio Terrasa, Santiago Esteban

**Affiliations:** 1 Hospital Italiano de Buenos Aires, Buenos Aires, Argentina.; 2 Universidad del Hospital Italiano de Buenos Aires, Buenos Aires, Argentina.; 3 Consejo Nacional de Investigaciones Científicas y Técnicas, Buenos Aires, Argentina.; 4 Instituto de Efectividad Clínica y Sanitaria, Buenos Aires, Argentina.

**Keywords:** Neoplasias del colon, Tamizaje masivo, Prevención secundaria, Sangre oculta, Colonoscopía, Argentina

## Abstract

Con el objetivo de evaluar la adherencia al tamizaje de cáncer colorrectal (CCR) de los afiliados al Plan de Salud del Hospital Italiano de Buenos Aires en Argentina, realizamos un estudio de cohorte retrospectivo con datos secundarios de la historia clínica electrónica. Se incluyó a todos los afiliados mayores de 50 años durante el período 2008-2022. Se evaluó el número y tipo de pruebas de tamizaje realizadas y la proporción de afiliados que se encontraba cubierta para el tamizaje. Se analizaron 112,112 participantes, con una mediana de edad de 58,6 años y un tiempo de seguimiento de 8,6 años. La prueba más utilizada fue la videocolonoscopía. El máximo de cobertura alcanzado fue 47,1% en diciembre de 2022. En conclusión, la adherencia al tamizaje de CCR fue subóptima, al igual que el método utilizado. Esta información puede ser utilizada para el diseño de una intervención multicomponente de mejora.

## INTRODUCCIÓN

El cáncer colorrectal (CCR) es una de las principales causas de morbimortalidad en el mundo y Argentina uno de los países latinoamericanos con mayor carga de enfermedad, con una incidencia y una mortalidad de 24,2 casos y 12,2 muertes cada 100,000 personas por año respectivamente ^1^^,^^2^.

El tamizaje de CCR, mediante la prueba de sangre oculta en materia fecal inmunoquímica (SOMF), es una estrategia eficaz y costo-efectiva para reducir la mortalidad por esta enfermedad ^3^^,^^4^. El Instituto Nacional del Cáncer (INC) de Argentina recomienda ofrecerlo a la población general entre los 50 y 75 años y considera como deseable una cobertura del 70% ^2^^,^^5^. Aunque el INC no recomienda la videocolonoscopía (VCC) como prueba de tamizaje en la población general, asociaciones como el Grupo de Trabajo Sobre Servicios Preventivos de los Estados Unidos (USPSTF por sus siglas en inglés) la aceptan como alternativa ^2^^,^^3^.

Hay pocos estudios publicados sobre la adherencia al tamizaje de CCR en la población argentina ^6^^-^^9^. Según la Encuesta Nacional de Factores de Riesgo, realizada en 2018, el 31,6% de las personas entre los 50 a 75 años se realizó este tamizaje alguna vez en su vida. Esta proporción aumenta en individuos con mayor nivel educativo o socioeconómico, con cobertura de la seguridad social o seguro privado de salud y en algunas jurisdicciones como la Ciudad Autónoma de Buenos Aires (CABA) ^6^.

El Hospital Italiano de Buenos Aires (HIBA) es una red formada por dos hospitales y quince centros ambulatorios ubicados en CABA y el Gran Buenos Aires (GBA). Funciona como prestador para seguros privados y obras sociales y además ofrece un seguro de salud (PS-HIBA) del cual es prestador único. Los afiliados al PS-HIBA son predominantemente de clase media. No contamos en la institución con un programa organizado de tamizaje de CCR. Si bien nuestro equipo ya publicó investigaciones vinculadas a esta problemática, no conocíamos la adherencia en la población del PS-HIBA a esta práctica ^10^^-^^13^. Por lo tanto, el objetivo de esta investigación es evaluar la adherencia de los afiliados al PS-HIBA al tamizaje de CCR.

**MENSAJE CLAVE**

Motivación para realizar el estudio. Es necesario contar con información sobre la adherencia de la población al tamizaje de cáncer de colon.

Principales hallazgos. La adherencia al tamizaje en el seguro de salud aumentó hasta alcanzar un máximo de 47,1% en diciembre de 2022, lo cual se encuentra por debajo de los objetivos deseables. El método más utilizado fue la colonoscopía.

Implicancias en salud pública. Esta información podría contribuir al diseño de una intervención multicomponente de mejora para la adherencia al tamizaje de cáncer de colon.

## EL ESTUDIO

Llevamos a cabo un estudio de cohorte retrospectivo con datos secundarios de historias clínicas electrónicas. Incluimos personas con edades entre 50 y 75 años, que hayan estado afiliadas por al menos un mes al PS-HIBA entre el 1 de enero de 2008 y el 31 de diciembre de 2022. Excluimos pacientes con datos faltantes o discordantes con respecto a su período de afiliación o con factores de riesgo para desarrollar CCR (antecedentes personales o familiares de CCR, pólipos colónicos, enfermedad inflamatoria intestinal, síndrome de CCR hereditario) o una colectomía total realizada antes de ingresar a la cohorte.

Se solicitó al Área de Gestión de la Información del Departamento de Investigación del HIBA un listado de las personas que cumplían con los criterios de inclusión, junto con sus fechas de nacimiento, afiliación al PS-HIBA, desafiliación o muerte, lugar de residencia y sexo. Se identificó la presencia de factores de riesgo mediante un *subset* de problemas asociados con factores de riesgo para cáncer de colon o colectomía cargados en la HCE (anexo 1). Solicitamos a la misma fuente información acerca de los estudios (SOMF o VCC) realizados por los participantes durante el seguimiento. Revisamos una muestra aleatoria de cien historias clínicas para comprobar la completitud y exactitud de la base de datos.

Los participantes ingresaban a la cohorte al momento que cumplían con los criterios de inclusión (edad mayor a 50 años o afiliación al PS-HIBA) y permanecían hasta su fecha de desafiliación, muerte, hasta que cumplían 75 años o bien hasta el fin del estudio (31/12/2022). El principal resultado de interés fue la cobertura para el tamizaje, es decir qué proporción de los participantes se encontraba cubierto al final de cada mes, por haber realizado una SOMF en el año previo o una VCC en los diez años previos.

Además, la cantidad de estudios de tamizaje que realizaron por año los afiliados del PS HIBA y la proporción de ellos que efectuó al menos un estudio durante el período de observación y la tasa anual de realización de estudios de tamizaje, dividiendo, para cada año, el total de estudios realizados por el número promedio de afiliados activos.

Como desenlaces secundarios, medimos la proporción de SOMF con resultado positivo (tasa de positividad), la proporción de VCC con resultados anormales (pólipos u otras lesiones precursoras de CCR) o preparación insuficiente, la proporción de pacientes con SOMF positiva que realizaron una VCC en los seis meses posteriores al resultado, la proporción de VCC precedidas por una SOMF positiva, y la proporción de solicitudes electrónicas de SOMF y de VCC que fueron efectivizadas por los participantes al cabo de seis meses (tiempo de vigencia de la orden).

Realizamos un análisis de subgrupos para evaluar si la cobertura de tamizaje al final del seguimiento se asociaba con la edad, el sexo, el año de ingreso a la cohorte o el lugar de residencia. Se utilizaron las pruebas de chi cuadrado y la prueba de tendencia no paramétrica de Cuzick para efectuar las comparaciones ^14^. Como análisis de sensibilidad, se consideró una cobertura de dos años para la SOMF. Para evaluar la proporción de VCC con resultados anormales, se analizó una muestra aleatoria simple de 100 colonoscopías. Todos los análisis y gráficos fueron realizados con R (R Foundation for Statistical Computing, Viena).

Este estudio fue realizado con datos secundarios anonimizados obtenidos de las historias clínicas electrónicas, en conformidad con el Código de Ética de la Asociación Médica Mundial (Declaración de Helsinki). Se obtuvo aprobación por parte del Comité de Ética de Protocolos de Investigación del HIBA (código 1522).

## HALLAZGOS

La base inicial incluía 114 763 participantes, de los cuales fueron excluidos 2637 (2,3%) por presentar factores de riesgo para CCR, 121 (0,1%) tener datos faltantes o contradictorios en sus períodos de afiliación y 14 por colectomía previa. El tamaño final fue 112,112 participantes.

En la Tabla 1 se presentan las características de la población de afiliados entre 2008 y 2022 al Plan de Salud del Hospital Italiano de Buenos Aires, Argentina. La mediana de edad al ingreso fue 58,6 años (rango intercuartílico 50,8 a 65,8) y la mediana de tiempo de seguimiento fue 8,6 años (rango intercuartílico 3,6 a 14,6). Se registraron 107,495 pruebas de tamizaje durante el período de observación, de los cuales 63603 (59,2%) correspondieron a VCC y 43892 (40,8%) a SOMF. En la Figura 1 se observa la evolución temporal de la realización de dichas pruebas. En el 2022, las tasas de realización de SOMF y VCC fueron de 21,8 y 105,3 estudios por cada 1000 afiliados por año, respectivamente. El 52,4% de los participantes se realizó al menos una prueba de tamizaje siendo la más utilizada la VCC en un 57,5% de los casos, seguida por una combinación de ambas pruebas en un 22,6% y por la SOMF en un 19,8%.

**Tabla 1:** Características de los afiliados entre 2008 y 2022 al Plan de Salud del Hospital Italiano de Buenos Aires, Argentina

Variable		n (%)
Sexo		
	Femenino	68397 (60,8)
	Masculino	44091 (39,2)
	Indeterminado	3 (0)
Lugar de residencia		
	Ciudad Autónoma de Buenos Aires	50572 (45,0)
	Partidos del Gran Buenos Aires	33433 (29,7)
	Resto de Argentina	1604 (1,4)
	Sin datos	26882 (23,9)
Edad de ingreso a la cohorte		
	50 años	25068 (22,3)
	Más de 50 años	87423 (77,7)

**Figura 1:** Pruebas de tamizaje de cáncer de colon efectuadas entre 2008 y 2022 por afiliados del Plan de Salud del Hospital Italiano de Buenos Aires en pacientes con edades entre 50 y 75 años.

SOMF: prueba de sangre oculta en materia fecal inmunológica. VCC: videocolonoscopia.

En la Figura 2 se observa la evolución de la cobertura en función del tiempo. En diciembre de 2022, 47,1% de los participantes se encontraba cubierto, lo cual representa el máximo valor de cobertura alcanzado durante el estudio. El sexo, la edad mayor a 60 años, el lugar de residencia en CABA o GBA, el año de ingreso a la cohorte antes de 2017 y la utilización de colonoscopía como método de tamizaje se asociaron con una mayor cobertura (Tabla 2).

**Figura 2:** Cobertura para tamizaje de cáncer de colón en función del tiempo Afiliados del Plan de Salud del Hospital Italiano de Buenos Aires. Período 2008-2022

SOMF: prueba de sangre oculta en materia fecal inmunológica. VCC: videocolonoscopia. Proporción de afiliados del Plan de Salud del Hospital Italiano de Buenos Aires con edad entre 50 y 75 años, que se encuentran cubiertos para el tamizaje de cáncer colorrectal al final de cada mes de seguimiento, por haber realizado una SOMF en el año previo o una VCC en los últimos diez años. Las líneas horizontales roja y dorada representan una cobertura del 40% y 70%, las cuales constituyen los objetivos considerados como aceptables y deseables, respectivamente, por el Instituto Nacional del Cáncer de Argentina. Para los primeros años de seguimiento, el nivel de cobertura puede estar subestimado, debido a que no se incluyeron en la base de datos estudios de tamizaje hechos con anterioridad a 2008.

**Tabla 2:** Cobertura para el tamizaje de cáncer de colon alcanzada en diciembre de 2022. Análisis de subgrupos.

Subgrupo		Participantes cubiertos para el tamizaje de cáncer de colon (%)	Participantes sin cobertura para el tamizaje de cáncer de colon (%)	Valor de p
Sexo				
	Masculino	9962 (46,0)	11686 (54,0)	0,001^e^
	Femenino	14846 (47,8)	16239 (52,2)	
Edad ^a^				
	50-55	2014 (24,1)	6350 (75,9)	0,001^f^
	51-60	4548 (43,1)	6006 (56,9)	
	61-65	5426 (51,2)	5180 (48,8)	
	66-70	5940 (54,3)	4990 (45,7)	
	71-75	6880 (56,0)	5399 (44,0)	
Edad (dicotómico)				
	50-60	6562 (34,7)	12356 (65,3)	0,001^e^
	61 o más	18.246 (54,0)	15569 (46,0)	
Lugar de residencia				
	CABA	11387 (49,1)	11828 (50,9)	0,001^e^
	GBA	7280 (45,7)	8666 (54,3)	
	Resto de Argentina	279 (40,3)	414 (59,7)	
Año de ingreso a la cohorte				
	2008-2017 ^b^	20717 (54,8)	17098 (45,2)	0,001^e^
	2018-2022	4091 (27,4)	10827 (72,6)	
Método de tamizaje utilizado ^c^				
	SOMF	946 (23,1)	3156 (76,9)	0,001^e^
	VCC	18369 (95,1)	944 (4,9%)	
	Combinación de SOMF y VCC	5493 (89,7)	628 (10,3)	
Completitud del seguimiento				
	Participantes con residencia en CABA o GBA, que ingresaron a la cohorte con 50 años y antes de 2017^d^	4660 (52,4)	4238 (47,6)	0,001^e^
	Resto de los participantes	2048 (46,0)	23687 (54,0)	

^a^ La edad se refiere a la edad en años al momento de la evaluación (diciembre de 2022). ^b^ Estos pacientes tienen al menos cinco años de seguimiento. ^c^ Se refiere a las pruebas de tamizaje utilizadas por los participantes durante todo el período de seguimiento, no necesariamente a la última prueba realizada. ^d^ Presentamos los resultados este subgrupo por separado ya que es menos probable que haya realizado estudios de tamizaje antes de ingresar a la cohorte (por la edad) y que los haya realizado por fuera de la red de atención del PS HIBA (por su lugar de residencia). Además, tiene un tiempo de seguimiento de al menos 5 años. ^e^ Prueba de chi cuadrado. ^f^ Prueba de tendencia no paramétrica de Cuzick. CABA: Ciudad Autónoma de Buenos Aires. GBA: Gran Buenos Aires. SOMF: prueba de sangre oculta en materia fecal. VCC: videocolonoscopía.

La tasa de positividad de SOMF fue 17,1%. De los participantes con SOMF positiva, 50,2% realizaron una VCC en los siguientes seis meses. Por otro lado, sólo el 12,6% de las VCC realizadas fueron efectuadas luego de una SOMF positiva.

El 43% de las VCC tuvo un resultado anormal (IC 95%: 33,1 a 53,2%) y el 4% tuvo una preparación inadecuada (IC 95%: 1,1 a 9,9%). De las solicitudes electrónicas de SOMF y VCC, el 52,5% y el 49,1% fueron efectivizadas durante los seis meses posteriores a la solicitud, respectivamente.

En el análisis de sensibilidad, si se considera una cobertura de dos años para la SOMF, la proporción de pacientes cubiertos para el tamizaje en diciembre de 2022 fue levemente mayor, con un valor de 48,4% (p<0,001).

## DISCUSIÓN

Los hallazgos evidencian que en diciembre de 2022 hubo una cobertura de 47,1%, la cual representa la máxima cobertura alcanzada dentro del período de estudio. La VCC fue el método más utilizado, con una tasa por año de 105,3 estudios por cada 1000 afiliados.

Durante el año 2020 se observa una marcada disminución de los estudios realizados, esto podría ser atribuible a las medidas de aislamiento por la COVID-19 ^15^. Durante el 2018 se observó además una disminución relativa de la realización de VCC, que podría explicarse por el fallecimiento de una conocida periodista argentina durante una endoscopía; mientras que en 2019 se produjo un ascenso en el número de SOMF, atribuible al reemplazo en nuestra institución del método de guayacol para para identificar sangre oculta en la materia fecal por la SOMF inmunoquímica, cuya realización es más sencilla para el paciente ^16^.

El nivel de cobertura alcanzado es inferior a la meta de 70% propuesta por el INC. Además, la prueba más utilizada es la VCC en lugar de la SOMF, que representa el método de elección según el INC ^2^^,^^5^.

Según la Encuesta Nacional de Factores de Riesgo de 2018, la proporción de personas de 50 a 75 años que se realizó alguna vez un tamizaje de cáncer de colon fue 51,3% en la CABA y el método más utilizado fue la VCC en un 42,6% de los casos, lo cual es similar a los resultados de nuestro estudio ^6^.

En cuanto a las causas de las bajas coberturas, en Argentina se publicaron dos investigaciones que señalan como barreras de acceso al tamizaje el bajo conocimiento de la población acerca del CCR y los métodos de tamizaje, en especial la SOMF, la baja percepción del riesgo y los tabúes culturales relacionados con el ano ^17^^,^^18^. Otros factores que pueden afectar la cobertura son las circunstancias económicas adversas, la debilidad o fragmentación del sistema de salud y la ausencia de un programa organizado de tamizaje que alcance a toda la población ^19^.

El estudio presenta limitaciones, no pudimos identificar a partir de nuestra base de datos si los pacientes participaron del tamizaje fuera del HIBA o antes del ingreso a la cohorte. Es poco probable que esto haya sucedido en el subgrupo de individuos que ingresan a la cohorte con 50 años y que residen en CABA o GBA, por lo que nuestra confianza en el cálculo de cobertura de este subgrupo es mayor. Por otro lado, no contamos con información acerca del motivo de realización de los estudios.

Sin embargo, de acuerdo a lo propuesto por Chubak y Hubbard consideramos a los pacientes como cubiertos, es decir sin necesidad de realizar nuevas pruebas de tamizaje, si realizaron una SOMF en el último año o una VCC en los últimos diez años, independientemente del motivo de realización de los mismos ^20^. Tampoco tuvimos en cuenta el resultado de los estudios en el cálculo de la cobertura. Si consideramos que el 17% de las SOMF son positivas y el 43% de las VCC tiene hallazgos anormales en la endoscopía, la cobertura real de los afiliados probablemente sea menor. Por último, al ser un estudio unicéntrico, nuestros resultados son poco extrapolables a la población general de Argentina u otros países de la región.

En cuanto a las fortalezas, dentro de nuestro conocimiento se trata del primer estudio que evalúa la adherencia al tamizaje de CCR en el subsistema privado de salud argentino. Incluimos a la totalidad de afiliados que pertenecían a la población objetivo según rango etario. El uso de datos de la HCE permite un registro y seguimiento longitudinal de los participantes.

A modo de conclusión, la cobertura para el tamizaje de CCR se encuentra lejos de los niveles deseables, a pesar de ciertas características favorables del PS-HIBA, como son el nivel socioeconómico de la población y la elevada disponibilidad de la VCC. Esta información puede ser útil como base para el diseño de una intervención multicomponente que contribuya a aumentar la adherencia al tamizaje de CCR, la cual debería enfocarse en el conocimiento y la accesibilidad de la SOMF tanto en pacientes como en profesionales de la salud.
